# Supplementary material for: Characterization of full-length and cytoplasmic tail-truncated envelope glycoproteins incorporated into human immunodeficiency virus (HIV-1) virions and virus-like particles
Source: J Virol. 2025 Nov 26;99(12):e01585-25. doi: 10.1128/jvi.01585-25 (PMC12724204; doi:10.1128/jvi.01585-25)
Supplement: Supplemental figures — Figures S1 and S2. [file jvi.01585-25-s0001.pdf]

SUPPLEMENTAL MATERIALS

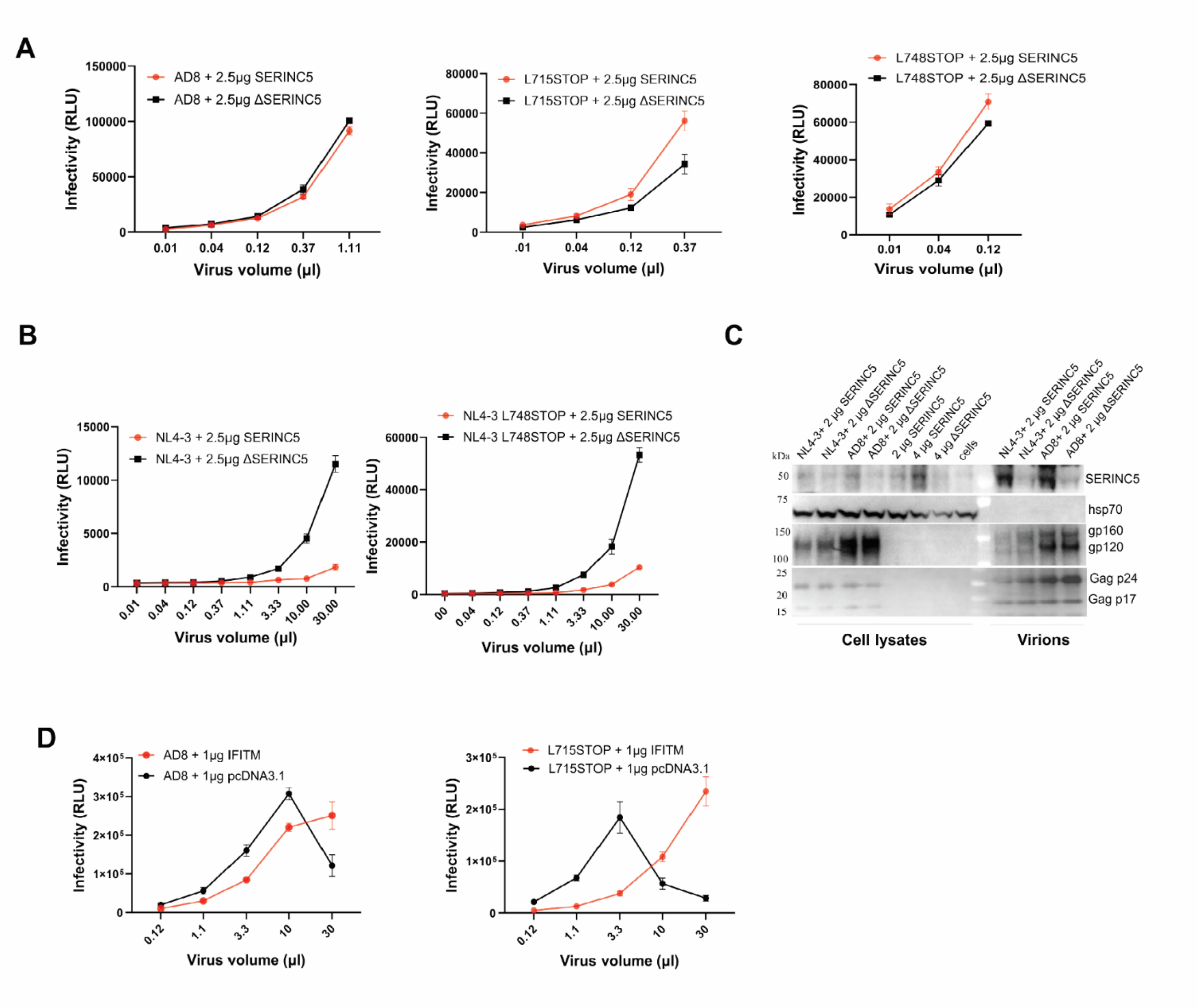

FIG S1 Susceptibility of pseudoviruses with Env variants to SERINC5 and IFITM restriction factors. (A) HEK 293T cells were transfected with pNL4-3 proviral constructs expressing the full-length HIV-1_{AD8} Env, the L715STOP Env or the L748STOP Env along with 2.5 μg of a plasmid encoding SERINC5 or a negative control plasmid ΔSERINC5. Forty-eight hours later, cell supernatants were cleared and incubated with TZM-bl cells. Two days later, the cells were lysed and luciferase activity was measured. (B) HEK 293T cells were cotransfected with pNL4-3 proviral plasmids encoding the wild-type HIV-$1_{NL4\text{-}3}$ Env or the NL4-3 L748STOP Env along with 2.5 μg of the SERINC5 or ΔSERINC5 plasmids. The infectivity of the produced virions was measured on TZM-bl cells, as described in A. (C) HEK 293T cells were transfected with proviral constructs expressing the HIV-1_{AD8} and HIV-$1_{NL4\text{-}3}$ Env variants along with the SERINC5 or ΔSERINC5 plasmids, as described in A and B above. In some experiments, the HEK 293T cells were mock transfected or transfected with the SERINC5 or ΔSERINC5 plasmids alone. Forty-eight hours after transfection, cell lysates and viruses were prepared and analyzed by western blotting for the indicated proteins. The western blots were developed with an anti-SERINC5 antibody (Abcam). (D) To study the effect of IFITM on HIV-1 infectivity, HEK 293T cells were transfected with pNL4-3 proviral constructs expressing the full-length HIV-1_{AD8} or L715STOP Envs along with 1 μg of a plasmid encoding IFITM or the pcDNA3.1 negative control. Forty-eight hours later, supernatants were cleared and added to TZM-bl cells. Two days later, the cells were lysed, and the luciferase activity was measured. For A-D, the results shown are typical of those obtained in at least two independent experiments.

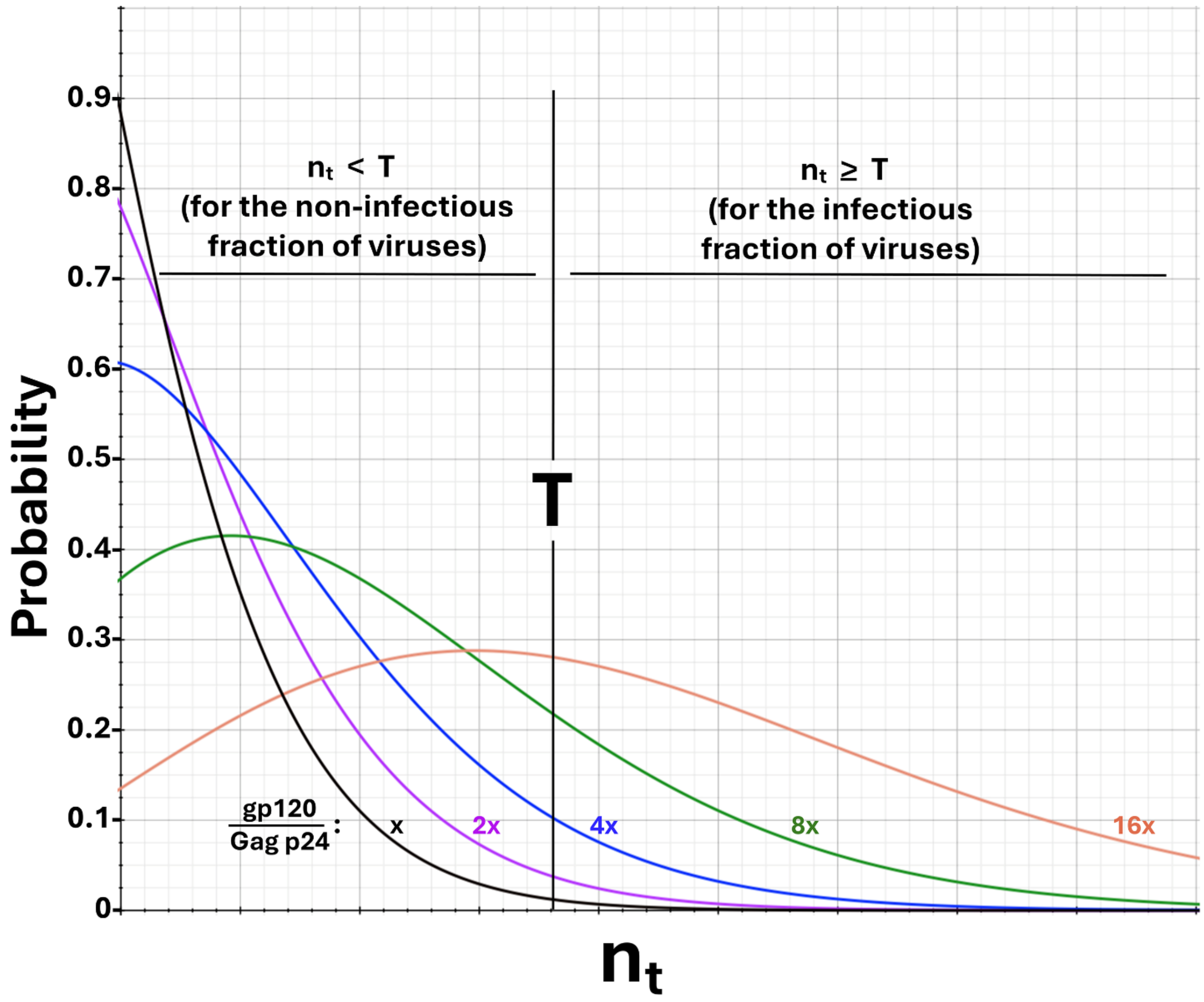

FIG S2 Theoretical distribution of Env trimers in a population of HIV-1 virions. The variable n_t is defined as the number of functional Env trimers on a virus particle that can potentially participate in the virus entry process. Assuming that Envs are sorted independently among virion particles, n_t will follow a Poisson distribution. Poisson probability distribution curves for n_t are shown for different average levels of cleaved Env in the virion population (gp120/Gag p24), expressed on an arbitrary scale (x, 2x, 4x, 8x and 16x). T is the number of Env trimers on a virion required for virus entry. On an infectious virus, n_t must be greater than or equal to T. HIV-1 preparations are known to exhibit low infectivity:particle ratios (115-118). The relationship between virion Env levels and infectivity observed in Figure 5C suggests that at most gp120/Gag p24 ratios achieved in our system, infectivity is limited by n_t. In this lower range of gp120/Gag p24 ratios, the Poisson distribution indicates that n_t will only be marginally greater than T on most infectious virions. When very high gp120/Gag p24 ratios are achieved by CT-truncated Envs, functional redundancy of virion Envs and increased resistance to virus neutralization may result.
